# Hybrid approach for ventral hernia associated with rectus diastasis: endoscopic single-port diastasis plication with open preperitoneal hernia repair. ENDOP technique in 10 steps

**DOI:** 10.1007/s00464-025-12540-4

**Published:** 2026-01-30

**Authors:** G. Bosch-Silvela, A. Bravo-Salva, M. Juvany-Gómez, M. Pérez-Guitart, A. Martínez-Solà, M. Jiménez-Gómez, L. Lorente-Poch, J. A. Pereira-Rodríguez

**Affiliations:** 1 Department of General Surgery, Abdominal Wall Surgery Unit, Hospital del Mar, Passeig Marítim 25-29, DC 08003 Barcelona, Spain; 2 MELIS Department, Universitat Pompeu Fabra (UPF), Barcelona, Spain; 3 Hospital del Mar Medical Research Institute (IMIM), Barcelona, Spain; 4 Abdominal Wall Surgery Member of Spanish Surgeons Association (AEC), Madrid, Spain

**Keywords:** Rectus diastasis (RD), Ventral hernia, ENDOP technique, Minimally invasive surgery (MIS), Hybrid approach, Endoscopic plicature

## Abstract

**Introduction:**

Rectus Diastasis (RD) associated with ventral hernia disrupts the fascia, complicating repair. The European Hernia Society (EHS) recommends linea alba plication with mesh repair. At our center, we usually repair only the hernia in asymptomatic low-to-moderate RD to minimize complications, opting for plication when quality of life or aesthetic concerns arise. We employ the ENDOP technique, a hybrid single-incision approach combining onlay ENDoscopic plication with sublay OPen mesh repair. To our knowledge, no prior technique has addressed both conditions using different abdominal wall layers. This study aims to demonstrate the procedure in 10 steps.

**Materials and methods:**

The technique is detailed step by step using the case of a 43-year-old female with primary umbilical hernia and moderate RD (T2D2H1), illustrated by a video showing the 10 steps.

**Results:**

Eleven patients underwent ventral hernia repair with ENDOP. Seventy-three percent were female, with a mean age of 55.4 years and BMI of 28.6. Mean hernia defect size was 1.64 cm and mean maximal transverse diastasis 5 cm. All but two patients were treated as same-day cases. No intraoperative complications occurred. One patient developed hematoma and three developed postoperative seroma (Clavien–Dindo I), none requiring intervention. Mean follow-up was 13 months.

**Discussion:**

The ENDOP technique allows simultaneous correction of two pathologies through one incision, fulfilling the principles of minimally invasive surgery. The ENDOP technique is reproducible and safe. It avoids peritoneal access and allows optimal preperitoneal mesh placement, preserving the retromuscular plane for potential future repair and providing effective bulging control, thereby improving both functional and aesthetic outcomes.

**Conclusions:**

The ENDOP technique may represent a valid option for managing this combined pathology.

**Supplementary Information:**

The online version contains supplementary material available at https://doi.org/10.1007/s00464-025-12540-4.

Rectus diastasis (RD) is defined as the abnormal separation of the rectus abdominis muscles exceeding 2 cm, leading to an expansion of the linea alba [1]. Unlike a hernia, RD does not represent a true abdominal wall defect and, in most cases, constitutes a non-acute condition far from posing a surgical emergency. However, it cannot always be considered a purely asymptomatic condition. Beyond its aesthetic impact on body image perception, RD has been associated with a variety of functional disorders. Musculoskeletal alterations include reduced core stability, pelvic instability, and lumbar back pain, which may impair daily activities or physical performance. In the urogynecological sphere, RD has been linked to urinary incontinence, pelvic floor dysfunction, and a higher incidence of prolapse-related symptoms [2–4]. Gastrointestinal complaints such as bloating or abdominal discomfort have also been reported, likely related to impaired abdominal wall mechanics. When RD coexists with a concomitant ventral hernia, the previously intact fascia becomes disrupted, transforming into a “diseased” midline, a situation that often modifies therapeutic decision-making.

There is limited consensus in the literature regarding the optimal management of this concomitant condition. According to the latest European Hernia Society (EHS) guidelines, “a mesh-based repair of rectus diastasis (RD) with concomitant midline hernias is suggested, while plication of the linea alba may be sufficient to repair a diastasis associated with very small (< 1 cm) umbilical or epigastric hernias” (KQ8) [1]. This recommendation, however, is supported by low-quality evidence and a weak grade of recommendation, reflecting the absence of comparative studies and the heterogeneity of techniques described. The guidelines do not establish a superior technique.

Numerous publications discuss their combined repair in a single surgical time through multiple minimally invasive (MIS) techniques, including intra-abdominal approaches such as IPOM + , LIRA, and Ventral-TAPP; as well as extraperitoneal methods like eTEP/FESSA, REPA/SCOLA/MILA and the stapled retrorectus repair described by Moore. Hybrid techniques combining open and MIS have also been reported, including ELAR plus/MILAR/EMILOS and stapled approaches as well as THT/miSAR [5–22].

None of them mixes different planes to simultaneously restore the hernia defect and diastasis recti, and some of them address RD using approaches similar to hernia repair, although evidence remains limited.

Although the literature on ventral hernia repair is extensive, only few studies have specifically addressed whether untreated rectus diastasis (RD) independently worsens long-term outcomes such as complications or recurrence when only the hernia is repaired [23–29]. In our clinical practice, to minimize the risk of complications associated with extensive dissection, we usually perform hernia repair alone in patients with asymptomatic, low-to-moderate rectus diastasis (D1–D2). However, in selected patients where RD has a demonstrable impact on quality of life—due to either functional symptoms or aesthetic concerns—plication of the diastasis may be considered. Importantly, the therapeutic decision is always individualized and made in agreement with the patient, since some prefer a simpler operation with faster recovery, while others prioritize the aesthetic outcome. This approach aims to balance the potential benefits of RD correction with the need to avoid unnecessary surgical morbidity.

We utilize a hybrid—open and MIS—and combined—onlay/sublay—technique. This procedure applies the most convenient approach for ventral hernia repair—the preperitoneal plane with mesh—and, without adding new incisions, we perform an endoscopic supra-aponeurotic plication through a single port: the ENDOP technique (ENDOscopic supra-aponeurotic plication with OPen preperitoneal repair).

In hernia repair, it is important to place the mesh in a sublay position, as studies have shown that this approach results in lower rates of surgical site infections (SSI) and recurrence compared to onlay techniques [30, 31].

On the other hand, RD is not considered a fascial defect itself, which means it is not necessary to use a large mesh implants—which are not without complications—to cover the entire supra-aponeurotic space. This hybrid technique, individualized to the patient who needs it, not only repairs this dual pathology but also allows the function and aesthetic appearance of the abdomen to be restored in a more than acceptable surgical time.

The aim of this study is to clearly and schematically demonstrate how our team approaches the ENDOP surgical technique in 10 steps through a single incision.

## Materials and methods

### Ethical approval

This study was approved by the institutional ethics committee (CEIM num.2024/11350/I), and all procedures were conducted in accordance with the principles outlined in the Declaration of Helsinki.

### Inclusion and exclusion criteria

#### Inclusion criteria

- Adult patients (≥ 18 years old) with primary midline small-to-medium ventral hernia (1–4 cm).
- Presence of rectus diastasis confirmed by clinical examination and CT-Scan.
- Elective surgical repair using the hybrid ENDOP technique.
- Availability of complete preoperative and postoperative data.

#### Exclusion criteria

- Recurrent, incisional, or lateral hernias.
- Obesity (IMC ≥ 35 kg/m.^2^) [32]
- Previous major abdominal surgery.
- Emergency surgical procedures.
- Lack of postoperative follow-up or incomplete records.
- Patients with contraindications for general anesthesia.

### Case description

**Fig. 1:**
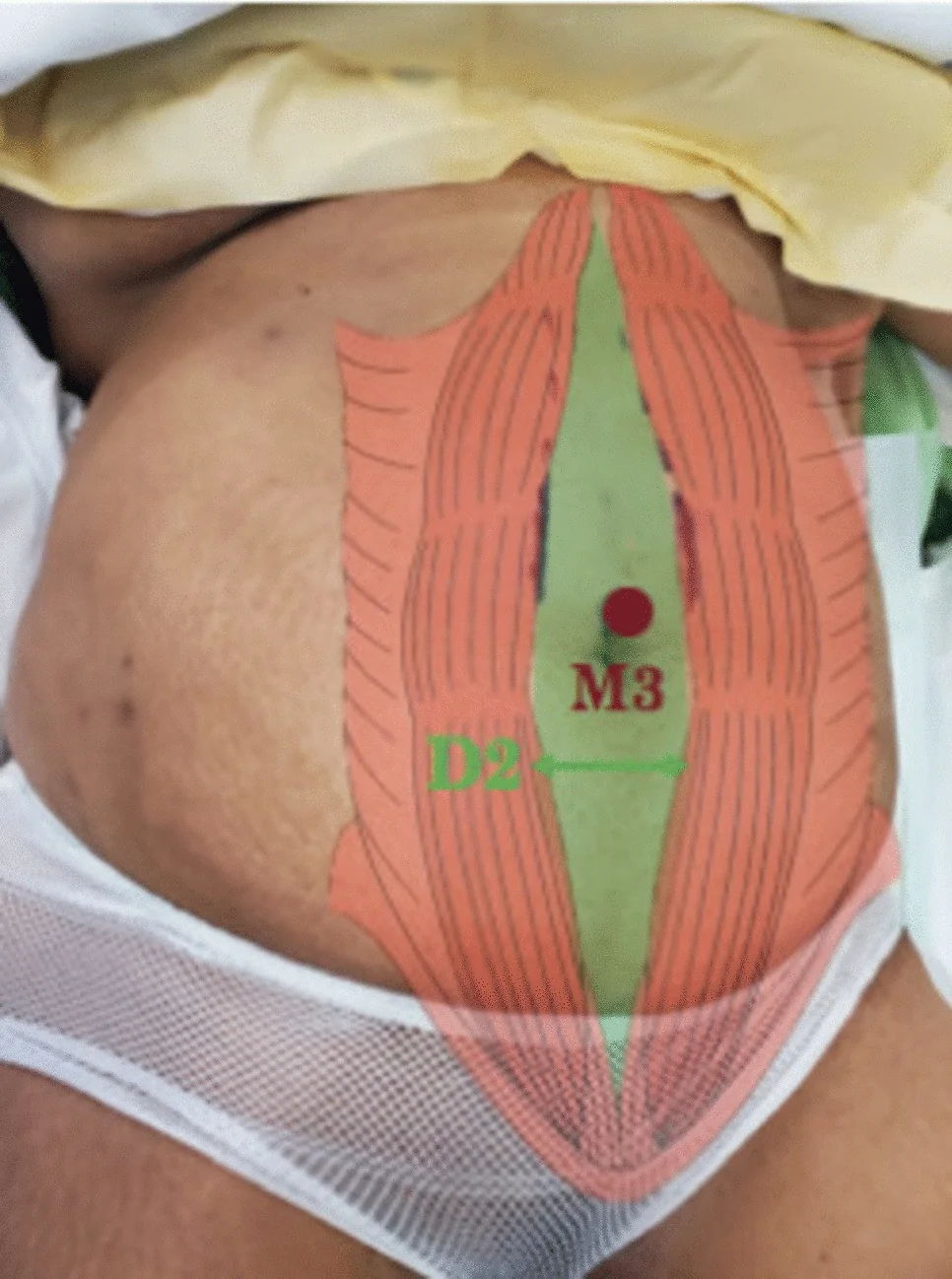
Preoperative physical examination findings. A globose abdomen is observed, predominantly due to subcutaneous adipose tissue, with a primary ventral hernia accompanied by rectus diastasis. Clinical examination reveals a midline herniary protrusion, along with medial separation of the rectus abdominis muscles, characteristic of diastasis

In this study, the technical description is based on the surgical treatment of a 43-year-old female patient—one of the patients from our case series—with no chronic diseases or prior abdominal surgeries. On physical examination, she presented with a primary ventral umbilical hernia concomitant to a moderate RD (Fig. 1). The patient also associated Type-I-obesity with a BMI of 31 (Fig. 1).

### Preoperative study

**Fig. 2:**
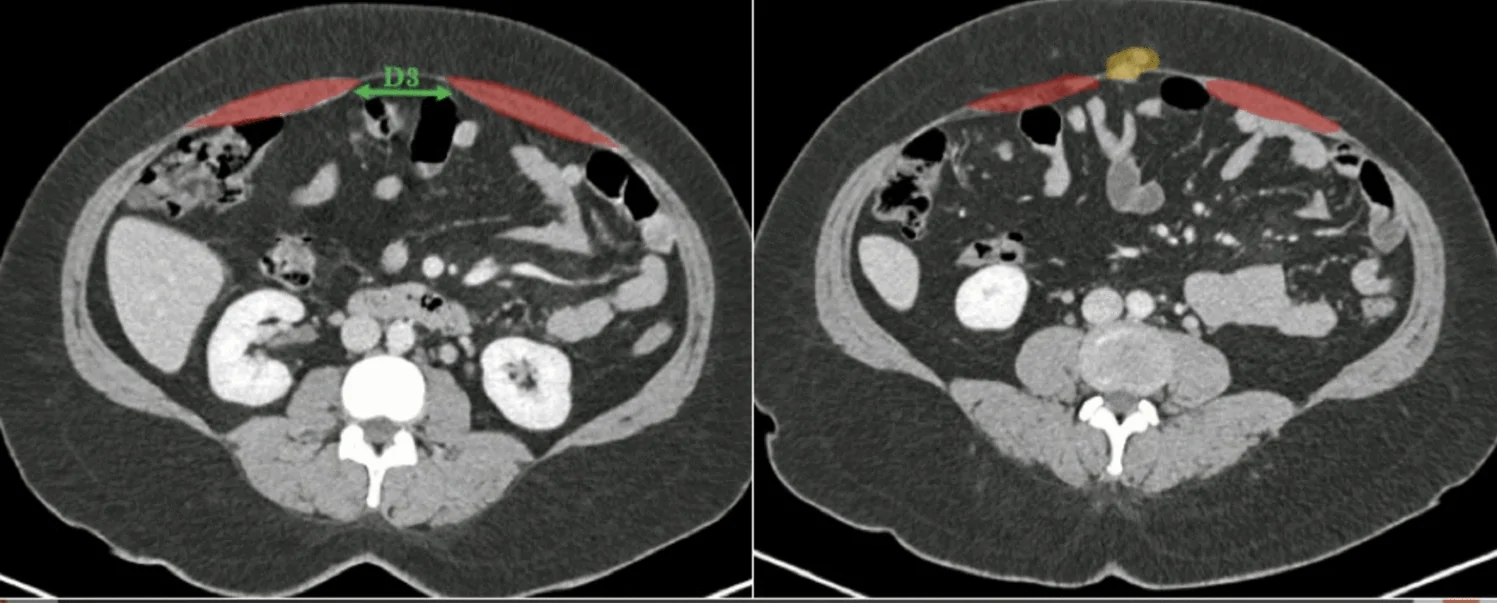
Preoperative computed tomography (CT) scan. Schematic illustration highlighting the two abdominal wall disorders present in the patient. The rectus diastasis is marked in green, showing the separation of the medial borders of the rectus abdominis muscles along the linea alba. The umbilical hernia is outlined in yellow, containing preperitoneal fat without signs of incarceration. This imaging modality provides a detailed anatomical evaluation, confirming the dual pathology and serving as a valuable tool for preoperative planning

Preoperative knowledge of anatomical structures is crucial for properly planning the repair route, playing a significant role in the success of this surgery. In the abdominal CT performed, we can observe a 5 cm diastasis and in the parallel illustration a small defect of 1 cm with fat content (Fig. 2). With these data and according to the EHS classification [1], it is a T2D2H1 case (Fig. 2).

**Fig. 3:**
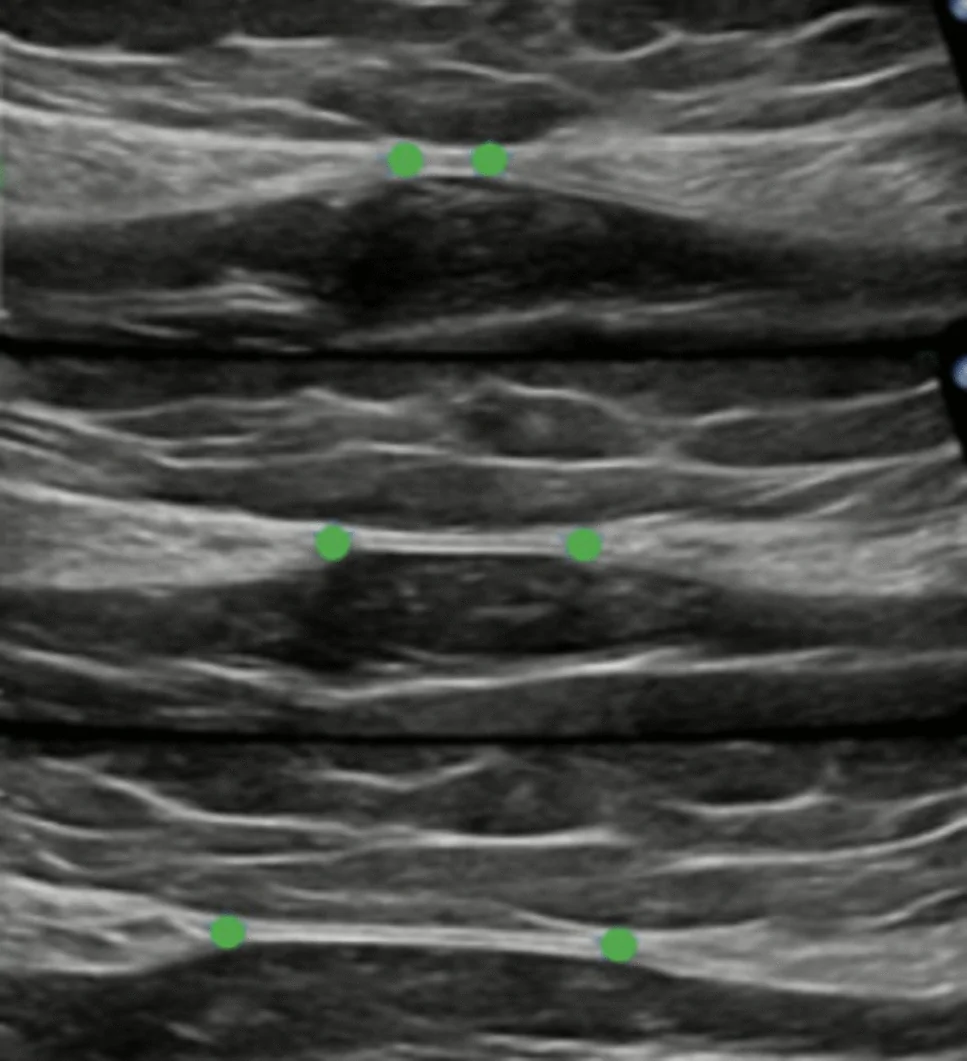
Intraoperative ultrasound image. The three standardized measurements used for evaluating the abdominal wall are highlighted in green. These include the width of the rectus diastasis at the supraumbilical, umbilical, and infraumbilical levels. Intraoperative ultrasound serves as a dynamic and precise tool for assessing the extent of the diastasis, marking it and guiding the surgical strategy, especially in cases involving minimally invasive or hybrid techniques

In the operating room, prior to initiating the procedure, ultrasound is performed to identify the medial borders of both rectus muscles. The RD width is measured at 3 points: subxiphoid, supraumbilical, and infraumbilical and the hernia defect is marked (Fig. 3).

### Surgical devices and materials

The following devices and materials were used during the procedures: Monomax® (B. Braun Surgical S.A., Rubí, Spain), Alexis® wound protector/retractor and GelPOINT® access platform (Applied Medical, Rancho Santa Margarita, CA, USA), Symmcora® barbed suture (B. Braun), Vicryl® and Stratafix® sutures (Ethicon, Johnson & Johnson, Somerville, NJ, USA), and the Ventralex™ Hernia Patch (Bard Davol Inc., Warwick, RI, USA).

### Single port and patient position in ENDOP hybrid approach

**Fig. 4:**
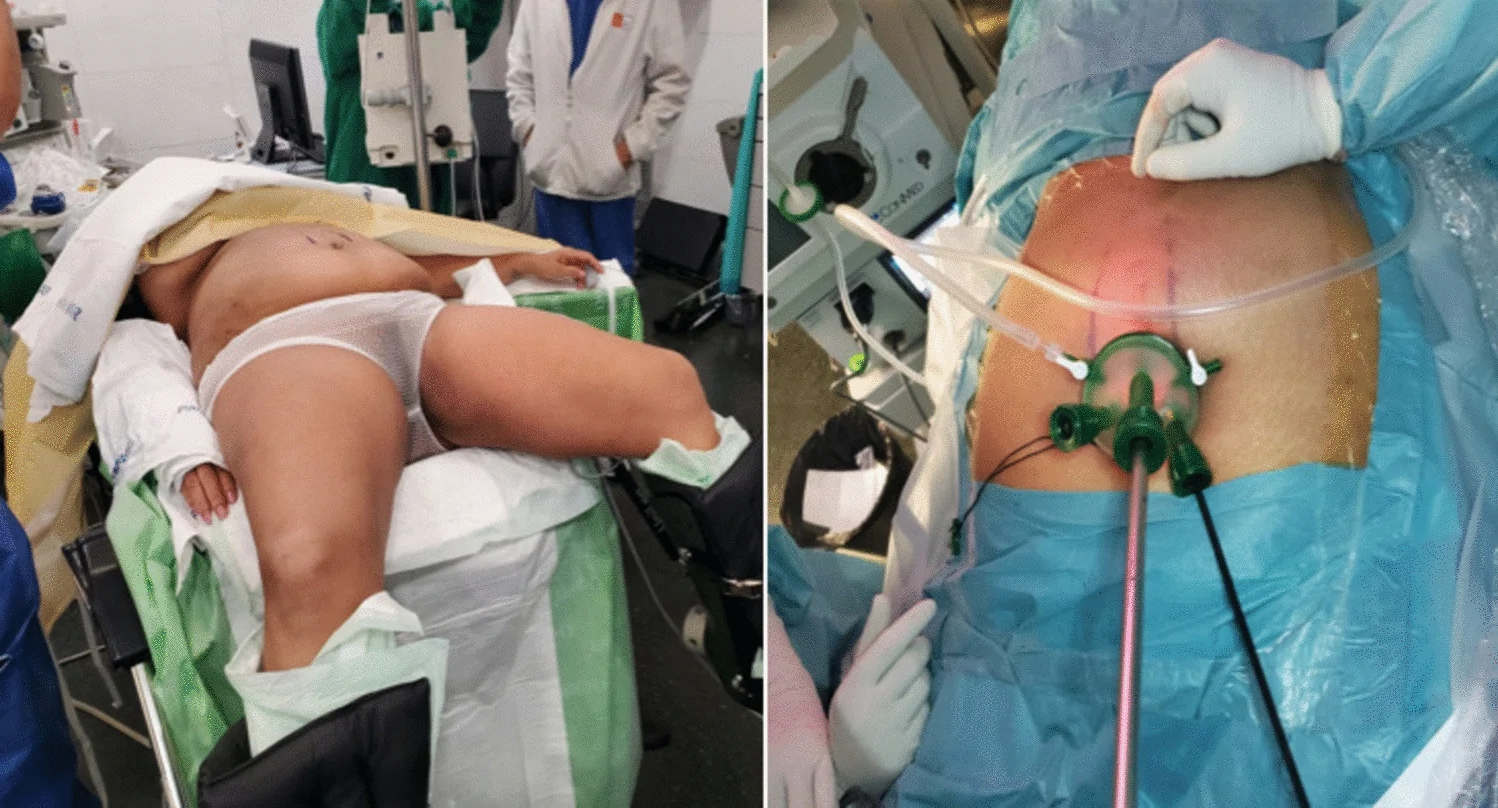
Left: the patient is in a supine position with a 10-degree anti-Trendelenburg tilt, legs apart, and arms tucked close to the body. The table can be tilted left/right if needed during the reparation, but normally not necessary. Right: placement of the single port centered around the umbilicus, through the previous hernia

To proceed with the surgical procedure, patients are positioned supine with lowered leg supports and the single port or trocar is placed through the previously open-repaired ventral hernia, as shown below (Fig. 4).

### ENDOP technique step by step

**Fig. 5:**
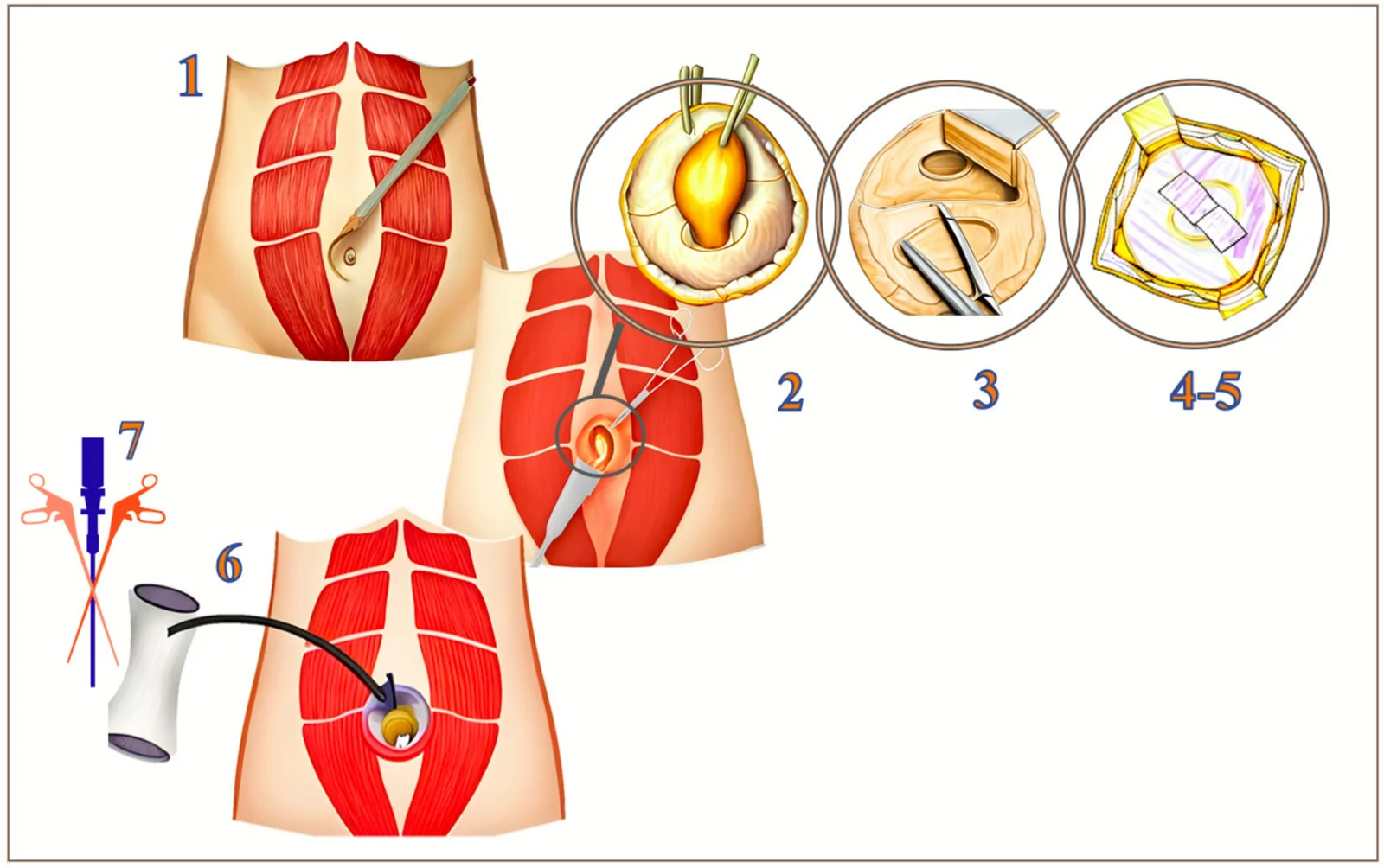
Surgical technique: Steps 1 to 7 of the hybrid ENDOP approach. **Step 1**. A 2-cm semicircular supraumbilical skin incision is performed after preoperative marking. **Step 2.** Dissection of subcutaneous tissue allows identification of the hernia and fascial planes. The hernia sac is carefully dissected circumferentially and reduced into the abdominal cavity. **Step 3.** Creation of the preperitoneal space is carried out beneath the fascia, forming a virtual dissection plane using blunt techniques (digital dissection, gauze) or scissors when needed. **Step 4.** A Ventralex mesh is inserted into the preperitoneal space and fully deployed to cover the defect adequately. **Step 5.** The mesh is fixated with a continuous suture using long-term absorbable monofilament (2–0 Monomax®), simultaneously achieving closure of the fascial defect. **Step 6.** The procedure then transitions to the endoscopic component for diastasis repair. A small Alexis® retractor is placed through the same incision to initiate supraponeurotic dissection. **Step 7.** A mini GelPOINT® single-port device is inserted through the existing incision, allowing for CO₂ insufflation (10–12 mmHg) and enabling endoscopic access to the retromuscular space for continued repair. This stepwise sequence illustrates the transition from an open preperitoneal hernioplasty to a minimally invasive approach for complete midline reconstruction in a single-port setting

**Fig. 6:**
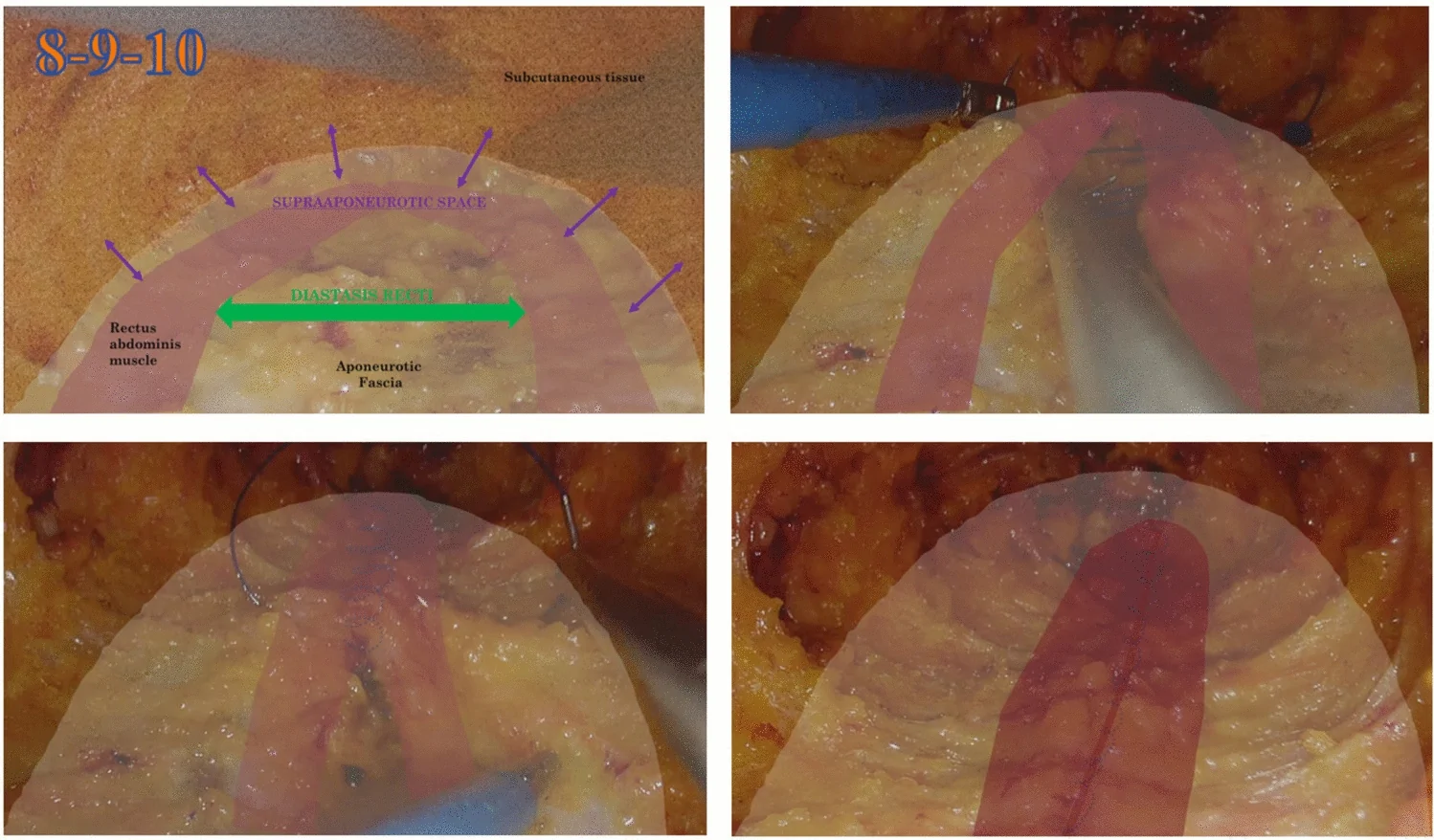
Surgical technique: Steps 8 to 10 – Endoscopic plication of rectus diastasis. **Step 8.** Dissection of the supra-aponeurotic space is initiated using electrocoagulation, creating a plane between the aponeurosis (inferior) and the subcutaneous tissue (superior). **Step 9.** The dissection is extended laterally beyond the full width of the diastasis, achieving an overlap of approximately 3–4 cm on each side. This lateral dissection is essential to prevent postoperative bulging and to ensure a smooth and aesthetically favorable contour. **Step 10.** Endoscopic plication of the rectus diastasis is performed using a continuous barbed monofilament long-term absorbable suture (0 Symmcora®)

**Fig. 7:**
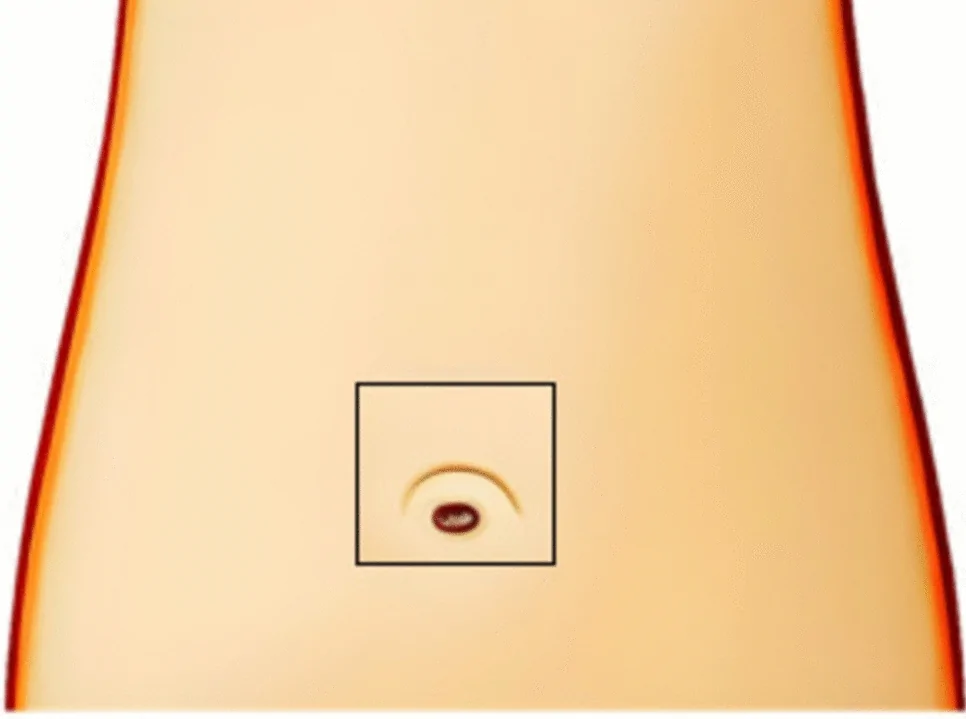
Final closure and immediate aesthetic result. The image illustrates the completion of the procedure with skin closure through the original 2-cm supraumbilical incision. This single-incision approach allows for the concomitant repair of two abdominal wall pathologies: a primary ventral hernia and rectus diastasis. The minimal access technique offers the advantage of reduced tissue trauma and favorable aesthetic outcomes, with the incision strategically concealed within the natural contours of the umbilical region

The procedure was divided into ten stages, focusing on some key points or critical spaces. This hybrid surgery consists of two clear phases: the open preperitoneal repair of the ventral hernia and the endoscopic plication of the diastasis. The surgery begins with the ventral hernia repair (steps 1–5) and is followed by the endoscopic diastasis repair (steps 6–10), (Figs. 5, 6, and 7).

1. **Incision:** After marking, a 2-cm semicircular skin incision is made along the supraumbilical margin.
2. **Hernia Sac Dissection and Reduction:** The subcutaneous tissue is dissected until the fascia and hernia are identified. In this step, the hernia sac is dissected up to the aponeurotic defect, exposing the fascial margins in 360º, and the sac is reduced.
3. **Preperitoneal Space:** After the reduction of the hernial sac, the creation of the preperitoneal space is performed; a virtual space located beneath the fascia that can be dissected digitally, with the help of a gauze, or if necessary, with surgical instruments such as scissors.
4. **Mesh Placement:** A Ventralex mesh is placed in the preperitoneal space and properly extended.
5. **Mesh Fixation and Closure of the Defect:** In this step, the mesh is secured with continuous suturing using a long-term absorbable monofilament suture (2–0 Monomax®), effectively closing the defect.
6. **Alexis**® **Placement:** Up to this point, the procedure follows the conventional approach for a preperitoneal open hernioplasty. Now, we proceed with the endoscopic part and the complete repair of the diastasis. The RD is addressed through an endoscopic supraponeurotic dissection. In this stage, an small-sized Alexis® is placed.
7. **Single-Port Position:** A mini GelPOINT® single-port device inserted through the same 2-cm hernia incisión after the Alexis® is placed and CO2 is applied at a pressure of 10–12 mmHg.
8. **Supra-aponeurotic Space Dissection:** In this step, dissection of the supra-aponeurotic space begins with electrocoagulation, leaving the aponeurosis below and the subcutaneous tissue above, as shown.
9. **Complete Diastasis Width plus Overlap Dissection:** This dissection continues until the medial supraponeurotic space is dissected to cover the entire width of the diastasis, with an overlap of 3–4 cm on each side. This overlap is crucial to avoid the bulging in the final result and achieve a good aesthetic result.
10. **Plication:** After measuring, we arrive at the final step: an endoscopic plication of the diastasis is performed using a continuous barbed monofilament long-term absorbable suture (0- Symmcora®). The correct positioning of the subcutaneous tissue and skin is also verified to prevent bulging. If bulging is observed, additional lateral dissection of 2–3 cm in the supraponeurotic plane is performed; however, this is not the case in this video. No supra-aponeurotic mesh is placed, and no drains are used as a routine.

The diastasis is fully repaired through the same single incision. As the suture approaches the umbilical region, where the Alexis® and GelPOINT® are located, laparoscopic angulation becomes uncomfortable and difficult. At that point, the GelPOINT® is removed while keeping the Alexis® in place, and the plication is completed via an open approach through the ring, without the cap.

Rectus diastasis is more common in the supraumbilical region and is rare infraumbilically [33–35]. Possible explanations include fewer crossing fibers reinforcing the linea alba supraumbilically [4], the upward distribution of intra-abdominal pressure during pregnancy, and added infraumbilical reinforcement from the Scarpa fascia and muscle insertions near the pubic symphysis [36]. When an infraumbilical component requires treatment, we just redirect the instruments and laparoscopic view and complete the plication below the umbilicus; starting from bottom to top with a separate running suture.

Subsequently, the single-port device is removed, and we proceed with the closure. The subcutaneous layer is closed using short-term absorbable sutures (3–0 Vicryl®), while the skin is closed with a continuous intradermal barbed suture made of short-term absorbable material (3–0 Stratafix®).

At this point, it is important to make some remarks in order to clearly differentiate ENDOP from other well-established techniques. The anterior rectus sheaths are not opened; instead, a supra-aponeurotic dissection is performed for direct vision plication. Techniques like REPA and SCOLA similarly avoid opening the anterior sheaths. Unlike them, ENDOP does not use a supra-aponeurotic mesh, trying to minimize seroma and mesh-related complications.

ENDOP preserves the retromuscular plane in most of the midline, an advantage in potential future hernia repairs. Current evidence supports that long-term absorbable sutures are comparable to non-absorbable sutures in terms of incisional hernia rates, with reduced chronic pain and fewer sinus formations. Studies have shown that long-term absorbable sutures such as PDS are effective and safe for rectus diastasis plication [37–41]. While non-absorbable sutures are durable, they carry greater risk of local complications. In our group, barbed sutures like Symmcora® and Darvin Loc® have yielded excellent results with no recurrences.

Although scientific evidence on abdominal binder use post-abdominal wall surgery is limited, some studies suggest benefits like reduced seroma, better pain management, and enhanced early mobility. Given the wide dissection in ENDOP and increased seroma risk, we generally recommend abdominal binder use for the first postoperative month, adjusting based on follow-up.

### Follow-up program

Patient data and follow-up were prospectively recorded in a REDCap database, which allowed systematic data collection, follow-up scheduling, and subsequent statistical analysis. Most patients were discharged on the same day of surgery under an ambulatory major surgery protocol. Postoperative follow-up consisted of an outpatient nurse wound check at 1 week, followed by surgeon visits at 1 month, 3 months, 6 months, 1 year, and 2 years.

## Results

A cohort of patients underwent ventral hernia repair using the ENDOP technique at our center. A total of 11 patients were included, with 73% being female. The mean BMI was 28.65, with one patient classified as normal weight, seven as overweight, and three as having obesity grade 1 as shown in Table 1. The mean transverse ventral hernia defect size was 1.64 cm, and the mean maximum transverse diastasis in the three standard measurement was 5 cm (Table 2).

**Table 1:** Demographic data of the cohort.

Sex	N	Age (years)	BMI (Kg/m^2^)
Female	8	52.6/50.5 (41–71)	28.21/27.35 (20.3–38.9)
Male	3	62.7/64.0 (53–71)	29.80/30.50 (27.1–31.8)
Overall	11	55.4/54.0 (41–71)	28.65/27.90 (20.3–38.9)

Demographic characteristics of the cohort stratified by sex, including number of patients, age, and body mass index (BMI) presented as mean/median (range)

**Table 2:** Hernia defect and rectus diastasis measurements

Measurement	Mean (cm)	Standard Deviation (cm)	N
Defect size	1.64	0.67	11
Subxiphoid RD	2.91	0.77	11
Supraumbilical RD	4.91	1.8	11
Infraumbilical RD	2.9	1.35	11

Mean, standard deviation, and sample size (n) of the hernia defect and rectus diastasis measurements at three anatomical levels: subxiphoid, supraumbilical, and infraumbilical

All patients, except two who faced social barriers, were treated as same-day cases and discharged on the day of surgery. All procedures were performed under general anesthesia. No intraoperative surgical complications occurred. The mean surgical time was 61 min (range: 32–93 min).

During the 90-day postoperative follow-up, three patient developed non clinical seroma (Clavien–Dindo I), which resolved without requiring treatment. Another patient who takes anticoagulant treatment also associated hematoma (Clavien–Dindo I) and another patient underwent a subsequent laparoscopic procedure and later developed an umbilical incisional hernia inferior to the previous hernia repair and diastasis plication. This was successfully repaired 36 months after the initial surgery. Patients cohort’s mean follow-up time is 13 months. No cases of readmission, reoperation, or postoperative mortality were recorded during the follow-up period.

## Discussion

As medicine advances, the pursuit of minimally invasive procedures that are not only effective but also accessible, cost-efficient, and conducive to rapid recovery—ultimately enhancing patients’ quality of life—becomes paramount. Yet, within this trend, a certain degree of overtreatment has emerged, particularly in the management of RD, with increasingly complex procedures being applied despite limited evidence regarding their true clinical relevance.

There is currently no clear evidence on the optimal management of RD. Traditionally, plastic surgeons have approached this condition through open abdominoplasty with “plication techniques” performed without mesh, reporting good outcomes in terms of quality of life, cosmetic results, and prevention of hernia development [2, 3, 15, 36–41]. With the increasing adoption of minimally invasive approaches, many general surgeons have extended the use of “hernia mesh repair techniques” to the treatment of RD. However, caution is warranted before generalizing these technically demanding procedures to all patients, particularly those with small- or medium-sized ventral hernias and low-to-moderate, clinically asymptomatic RD.

In the context of RD associated with ventral hernia, the EHS guidelines (KQ8) suggest plication of the linea alba with mesh repair, although this recommendation is supported by low-quality evidence and a weak grade of recommendation, as comparative studies are lacking [1]. The guidelines do not establish a superior technique but note that endoscopic subcutaneous dissection with plication and onlay mesh has been most frequently reported. Conversely, in the ventral hernia section, preperitoneal or intra-abdominal planes are recommended for mesh placement [1]. In line with this, ENDOP technique combines linea alba plication with preperitoneal mesh positioning, thus avoiding the need for large onlay meshes covering the entire supra-aponeurotic space. This hybrid approach aims to provide a more anatomical and less aggressive repair, minimizing foreign body reaction and reducing the extent of dissection.

The ENDOP hybrid technique—which involves the concurrent repair of two pathologies through a single incision—offers a balanced alternative, aligning with the main principles of minimally invasive surgery while avoiding excessive surgery for small-to-moderate defects (1–4 cm) and low-to-moderate RD (D1–D2). From our perspective, the ideal candidate is a patient with a primary small-to-medium ventral hernia, in whom an open preperitoneal repair would typically be indicated, and who, when concomitant RD is present, can be treated endoscopically through the same incision and surgical time, without the need for more aggressive procedures.

In this regard, and for selected cases, one of the main advantages of this technique is its less invasive nature compared to other traditional laparoscopic techniques, which require multiple incisions and are indicated for larger defects or more complex hernias [5–22]. This decline in the number of wounds not only minimizes surgical trauma and improves aesthetic outcomes but also decreases the risk of postoperative complications, primarily SSI and hematoma. Furthermore, the placement of the prosthesis in the preperitoneal or sublay plane and the midline plication adhere to the latest recommendations from the European Hernia Society (EHS) guidelines [1], while preserving the retromuscular space. This preservation is crucial, as it allows for the availability of this plane in case of recurrence and the need for reintervention, adding an additional level of safety for the patient.

The technique also offers control of the bulging in situ, ensuring a good immediate aesthetic result without having to rely on the definitive outcome improving in the first few postoperative months. Although its potential functional implications remain unknown and would require long-term follow-up studies, it positively impacts patient perception and satisfaction—key indicators of procedural success—and contributes to a more favorable emotional recovery [3, 4].

Finally, the tailored approach of this technique indicated on small ventral hernias with low-to-moderate RD allows patients to undergo surgery without the need for drains, simplifying the recovery after surgery and enabling a quick discharge from the hospitals under Ambulatory Major Surgery (AMS) regimes. The possibility for patients to return home on the same day not only enhances their comfort but also optimizes resource use within the healthcare system.

Regarding the learning curve, we consider that ENDOP technique does not pose substantial additional complexity for abdominal wall surgeons already familiar with the preperitoneal approach. The only added step is the endoscopic plication of the linea alba, which is technically straightforward once the dissection has been completed. For this reason, the procedure appears highly reproducible and likely associated with a relatively short learning curve, although future studies with larger case series would be necessary to objectively confirm this impression.

## Conclusions

Rectus diastasis has evolved from being a purely aesthetic condition to being recognized as an essential component of functional repair, thereby enhancing patients’ quality of life (2–4). During the last few years and with the emergence and development of MIS on abdominal wall procedures, a big number of surgical procedures have been published in order to repair both the rectus diastasis and ventral hernia. Nevertheless, current guidelines still reflect limited evidence and a lack of high-quality studies to determine the optimal approach for repairing this concomitant pathology.

ENDOP is a hybrid technique which combines both open and MIS and both supra-aponeurotic and preperitoneal layers, leaving the mesh in the preperitoneal space. It presents an effective surgical approach for the treatment of ventral hernias associated with rectus diastasis. The advantages are clear: it avoids access to the peritoneal cavity, preserves the retromuscular space for potential use in case of recurrence, and allows for effective control of the bulging in situ, thus providing correct functional and aesthetic outcomes.

In conclusion, ENDOP is not only a safe and feasible surgical technique, but it also presents multiple advantages that establish it as a valid option for managing this dual pathology.

## Supplementary Information

Below is the link to the electronic supplementary material.

Supplementary file1 (MP4 453320 KB)
