# Correction: Hybrid approach for ventral hernia associated with rectus diastasis: endoscopic single-port diastasis plication with open preperitoneal hernia repair. ENDOP technique in 10 steps

**DOI:** 10.1007/s00464-026-13389-x

**Published:** 2026-09-21

**Authors:** G. Bosch-Silvela, A. Bravo-Salva, M. Juvany-Gómez, M. Pérez-Guitart, A. Martínez-Solà, M. Jiménez-Gómez, L. Lorente-Poch, J. A. Pereira-Rodríguez

**Affiliations:** 1 Department of General Surgery, Abdominal Wall Surgery Unit, Hospital del Mar, Passeig Marítim 25-29, DC, 08003 Barcelona, Spain; 2 MELIS Department, Universitat Pompeu Fabra (UPF), Barcelona, Spain; 3 Hospital del Mar Medical Research Institute (IMIM), Barcelona, Spain; 4 Abdominal Wall Surgery Member of Spanish Surgeons Association (AEC), Madrid, Spain

## Correction to: Surgical Endoscopy (2026) 40:2661–2670 **https://doi.org/10.1007/s00464-025-12540-4**

The original online version of this article was revised to indicate that Symcora® suture was manufactured by B. Braun.

The original article has been corrected.

